# Regulation of aromatase expression in breast cancer treated with anastrozole neoadjuvant therapy

**DOI:** 10.3892/etm.2012.878

**Published:** 2012-12-24

**Authors:** CHIARA GHIMENTI, MAURIZIA MELLO-GRAND, ENRICO GROSSO, MARIA SCATOLINI, LEA REGOLO, ALBERTO ZAMBELLI, GIOVANNA CHIORINO

**Affiliations:** 1 Cancer Genomics Laboratory, Fondazione Edo ed Elvo Tempia Valenta, Biella;; 2 Units of Senology, IRCCS Fondazione ‘Salvatore Maugeri’, Pavia, Italy; 3 Medical Oncology, IRCCS Fondazione ‘Salvatore Maugeri’, Pavia, Italy

**Keywords:** breast neoplasms, aromatase inhibitors, genetic polymorphism, aromatase targeting microRNAs, intrinsic resistance

## Abstract

Aromatase inhibitors (AIs), such as anastrozole, are established in the treatment of hormone-dependent breast cancer. However, ∼20% of patients with hormone receptor-positive breast tumors treated with anastrozole do not respond and it remains impossible to accurately predict sensitivity. Since polymorphisms in the aromatase gene may influence the response to inhibitory drugs, we evaluated the presence of rs6493497 and rs7176005 polymorphisms (mapping in the 5′-flanking region of the CYP19A1 gene coding for the aromatase protein) in a cohort of 37 patients with postmenopausal breast cancer who received three-month neoadjuvant treatment with anastrozole. We then investigated any association of the polymorphisms with changes in aromatase mRNA expression change and/or response to treatment. We also analyzed five miRNAs computationally predicted to target aromatase, to observe any association between their expression and sensitivity to anastrozole. Three samples carried the two polymorphisms and the remaining samples were wild-type for both, however, no association with response or with aromatase mRNA basal expression level or expression difference after therapy was observed. Polymorphic samples that were resistant to anastrozole showed no change or decrease in aromatase expression following AI treatment, whereas an increase in expression was observed for the polymorphic responsive samples. No statistically significant correlation was observed between miRNA and aromatase mRNA expression, or with response to anastrozole neoadjuvant treatment. These data indicate that the polymorphisms analyzed are not involved in aromatase activity and that other epigenetic mechanisms may regulate aromatase protein expression.

## Introduction

Aromatase is encoded by the CYP19A1 gene, belongs to the cytochrome P450 superfamily and is a rate-limiting enzyme in the conversion of androgens into estrogens. Aromatase inhibitors (AIs), such as anastrozole, are extensively utilized in the treatment of estrogen receptor (ER)^+^/progesterone receptor (PgR)^+^ postmenopausal breast cancer patients, as AIs block paracrine production of growth stimulating hormones at the tumor-cell level (1,2). Despite proven clinical efficacy, such therapies are still associated with non-response in ∼20% of cases. To date, it remains impossible to accurately predict the response to such treatments.

Changes in aromatase mRNA or protein expression may have a role in the response of patients to AI therapy, and control of gene/protein expression or activity may be exerted at different levels.

It has been reported that the presence of two tightly linked single nucleotide polymorphisms (SNPs), rs6493497 and rs7176005, in the 5′-flanking region of the CYP19A1 exon 1.1 is associated with greater decrease in aromatase activity following anastrozole neoadjuvant treatment in breast cancer (3). The same SNPs are also associated with higher plasma estradiol levels in pre-AI and post-AI therapy patients and with higher basal aromatase activity in tumor samples (3).

In addition, miRNAs have a crucial role in gene regulation and changes in their expression may be associated with response to anticancer therapies. For example, expression of let-7f, an miRNA that targets CYP19A1, has been observed to increase after letrozole treatment in MCF-7 cells (4).

The aim of this study was to identify whether polymorphisms in the 5′-flanking region of the CYP19A1 exon 1.1 and/or the expression of miRNAs predicted to target the aromatase transcript are associated with aromatase RNA expression or response to three-month neoadjuvant anastrazole treatment in a cohort of patients with postmenopausal breast cancer.

## Materials and methods

### Patients

The tumor samples and clinical data were collected with the approval of the ‘Fondazione S. Maugeri’ ethics committee and informed consent from the patient. The 37 patients enrolled into this study between July 2004 and November 2007, were postmenopausal and had breast cancer of stage T2 or T3, diameter >2.5 cm, any lymph node status and no distant metastasis (5). The tumors were HER2/neu^−^ and ER^+^/PgR^+^, with the exception of two that were ER^+^/PgR^−^(5). The patients received neoadjuvant therapy with anastrozole (Arimidex™; AstraZeneca, London, UK) 1 mg/day PO for three months. The clinical response was evaluated by serial tumor clinical examination and mammary ultrasound bidimensional measurements, performed by a single operator prior to, during and following treatment. Patients with a reduction in tumor volume of ≥30%, according to RECIST criteria (6) were classified as responders (Rs).

### Sequence analysis

Total DNA was extracted from 37 formalin-fixed, paraffin-embedded breast cancer biopsies using High Pure PCR Template Preparation kit (Roche Molecular Biochemicals, Basel, Switzerland), according to the manufacturer’s instructions.

For dye-labeled terminator sequencing, DNA samples were amplified by PCR for rs6493497 and rs7176005 polymorphic sites in the 5′-flanking region of CYP19 exon 1.1. The oligo-nucleotide sequences are listed in Table I. PCR was carried out in a reaction volume of 25 *μ*l containing ∼100 ng genomic DNA, 50 mM KCl, 10 mM Tris-HCl pH 8.3, 1.5 mM MgCl_2_, bovine serum albumin (BSA 1 ng/*μ*l, 200 *μ*M dNTPs, 0.2 *μ*M primer and 0.1 U/*μ*l Taq polymerase. Amplification consisted of an initial denaturation at 95°C for 5 min, followed by 35 cycles of 1 min at 95°C, 1 min at 58°C and 1 min at 72°C, with a final extension at 72°C for 7 min.

PCR products were purified using the Wizard SV Gel and PCR Clean-Up system (Promega, Madison, WI, USA) and sequenced using a Big Dye Terminator V3.1 Cycle Sequencing kit and AB 3500Dx DNA Analyzer (Applied Biosystems, Foster City, CA, USA). All the sequencing data were analyzed by Variant Report 1.1 (Applied Biosystems).

### Gene expression profiling

Gene expression profiling and data analysis of 17 Tru-cut biopsies and 13 matched surgical resections were performed as previously described (7). Aromatase mRNA expression was retrieved from the matrix containing the log_2_ of the ratios of the expressions of each gene in the breast samples and in the Human Universal Reference (Clontech, Palo Alto, CA, USA). Three different probes detected the aromatase gene in the Agilent 4x44k Whole Human Genome Oligo Microarray kit (Agilent Technologies, Palo Alto, CA, USA). The differences in aromatase expression between the Tru-cut and post-operatory samples were calculated.

### Reverse transcription-quantitative polymerase chain reaction (RT-qPCR) of CYP19A1

Total RNA was extracted from formalin-fixed, paraffin-embedded breast cancer pre-treatment and post-surgical biopsies using Recover All Nucleic Acid Isolation kit (Ambion Inc., Austin, TX, USA) according to the manufacturer’s instructions.

RT-qPCR was performed as previously described (7) to analyze CYP19A1 expression using commercial primers (Hs_CYP19A1_2_SG QuantiTect Primer assay; Qiagen, Duesseldorf, Germany). First strand cDNA synthesis was performed using 200 ng total RNA. Data were presented as log_2_ fold change of CYP19A1 expression in samples relative to the Universal Reference, after normalization with an endogenous control (RPLP0).

### miRNA target prediction analysis

MiRanda, PITA, TargetScan and microCosm algorithms were used for the identification of putative miRNAs that target aromatase mRNA. miRNAs predicted by at least two algorithms were chosen for further analysis (miR-661, miR-1183, miR-1207-5p and miR-98). miR-378 was selected due to its known role in targeting aromatase in pigs (8).

### RT-qPCR of miRNA

miR-378, miR-661, miR-1183, miR-1207-5p and miR-98 were evaluated in 29 samples, from which total RNA from pre-treatment biopsies was extracted. Reverse transcription and amplification were carried out according to the TaqMan Small RNA assay protocol (Life Technologies, Austin, TX, USA). RNU48 was selected as an example of ‘housekeeping’ miRNA.

## Results

### Patient classification

Using the response criteria, 19 patients were classified as Rs and 18 as non-responders (NRs) (5). Only one patient (10050) showed disease progression during the treatment.

### Aromatase polymorphism analysis

Of the 37 genomic DNAs, 29 were successfully sequenced for the rs6493497 polymorphism, showing the presence of the variant base in three samples (10028, 10043 and 10056). Thirty-three out of 37 genomic DNAs were successfully sequenced for the rs7176005 polymorphism, showing the presence of the variant base in the same three samples (10028, 10043 and 10056). However, two of the three polymorphic samples were NR (10028 and 10056), whereas one (10043) showed 39% response.

### Aromatase expression analysis

Aromatase expression values from gene expression profiles were available for 13 biopsies and corresponding surgical resections (5). RT-qPCR values were available for 3 further pre/post treatment pairs (Table II).

CYP19A1 expression differences following treatment are listed in Table II. Sample 10056 (NR) showed a decrease in aromatase expression, whereas for sample 10028 (NR) the difference between pre- and post-treatment expression was negligible. Sample 10043 (R), however, showed the second highest positive differential value and the highest among R patients.

Prior to treatment, the aromatase expression level in sample 10056 (NR) was the second highest among all the patients and the highest among NRs, while sample 10043 (R) showed the lowest expression level before treatment among all the patients. Pre-treatment sample 10028 (NR) expression, however, was close to the average (Table III).

### Aromatase miRNA analysis

The correlation was calculated between miRNA expression and response to therapy or aromatase expression. No correlation was observed between miRNAs and therapy response.

Differential expression was evaluated for each miRNA in R versus NR patients. The overall mean expression level was higher in the Rs than in NRs for miR-98, miR-1183 and miR-1207, while it was lower for miR-378 and miR-661, with no statistically significant results for any of the miRNAs (P>0.05; Table IV).

### Statistical analysis

Class comparison between R and NR was performed using an unpaired Student’s t-test. P<0.05 was considered to indicate a statistically significant result.

## Discussion

Colomer *et al*(9) identified an association between the presence of the rs4646 single-nucleotide polymorphism in the 3′ untranslated region of the aromatase gene and efficacy of the AI letrozole, in terms of complete response rate and time to progression in 67 HR^+^ (hormone-receptor positive) advanced breast cancer postmenopausal patients. This polymorphism was also observed to be associated with poor response to letrozole neoadjuvant treatment and to poor outcome in elderly HR^+^ postmenopausal patients (10).

More recently, three single nucleotide polymorphisms of CYP19A1 (rs700518, rs10459592 and rs4775936) were demonstrated to predict clinical outcome and adverse events associated with letrozole treatment in patients with metastatic breast cancer (11).

In another study, rs6493497 and rs7176005 polymorphisms were shown to induce a very great decrease in the aromatase enzymatic activity following AI neoadjuvant treatment and to influence the ability of regulatory proteins to bind DNA at the CYP19A1 promoter (3). On the basis of these findings, the authors hypothesized that rs6493497 and rs7176005 polymorphisms may also affect aromatase transcriptional regulation (3).

The present study of 37 postmenopausal breast cancer patients confirms the close association between the two polymorphisms, as the two variant sequences were observed in the same samples. However, our data are unable to clarify the expression modulation. One (sample 10056) of three polymorphic samples confirmed the hypothesis of Wang *et al*(3), showing the second highest pre-treatment expression level among all the patients (the highest among NRs) and a negative expression difference after neoadjuvant therapy, which may be considered an indicator of a reduction in aromatase transcription. Sample 10028 did not show a high basal expression and the difference between pre- and post-treatment was negligible. In disagreement with the hypothesis of Wang *et al*(3), sample 10043 showed the second highest increase in aromatase expression (the highest among Rs) and the lowest basal expression level among all the samples. Therefore, the presence of the two SNPs does not appear to be associated with response to treatment, nor with aromatase transcriptional modulation. The R samples showed an increased aromatase expression, whereas aromatase expression decreased or did not change in NRs.

In addition, the greatest reduction of expression between pre- and post-treatment specimens occurred in sample 10013, classified among Rs and showing a wild-type sequence. Furthermore, samples 10051 and 10054 show the highest increase in aromatase expression among NRs, suggesting that resistance to anastrazole is achieved by alternative mechanisms that are independent of CYP19A1 expression modulation.

In a previous study by our group (7), gene expression profiles prior to and following therapy were used to investigate differences in expression changes between Rs and NRs and to identify a gene expression signature associated with response to therapy. Notably, unsupervised clustering analysis, using the genes with higher gene expression changes after anastrozole treatment, classified 10028 and 10056 samples within the R group, although their percentage of response was <30%. By contrast, the 54-gene signature was able to predict response in 100% of cases, indicative that the molecular features of the tumors are stronger determinants of response than the presence of the two investigated polymorphisms.

We also investigated any difference at the global gene expression level between polymorphic and non-polymorphic samples. However, due to the small size of the samples, we were unable to identify critical differentially expressed genes or remarkable metabolic processes between the two groups.

Since the cross-talk between miRNAs and protein coding genes often represents a fine regulation circuit in the response to numerous anticancer therapies, we analyzed the expression levels of five miRNAs predicted to target aromatase (miR-378, miR-661, miR-1183, miR-1207-5p and miR-98). No statistically significant correlation was observed with aromatase mRNA expression nor with response to anastrozole neoadjuvant treatment.

These data indicate that the polymorphisms analyzed are not involved in aromatase activity. However, epigenetic mechanisms that regulate aromatase protein expression deserve further investigation and may be taken into account to select patients for anastrozole neoadjuvant treatment.

## Figures and Tables

**Table I.** PCR primer sequences for CYP19A1 (aromatase) polymorphism amplification.

Polymorphism	Primer name	Primer sequences (5′-3′)
CYP19A1 rs7176005	717F	TCTCTCAGCAATACCCACCA
CYP19A1 rs7176005	717R	CCACCACACACCACATTGTT
CYP19A1 rs6493497	649F	CATTCCAGAGGAGGTCATGC
CYP19A1 rs6493497	649R	AGTTTCTGGAGGGCTGAACA

**Table II.** Difference between CYP19A1 expression (log_2_ ratio of sample with respect to Human Universal Reference Total RNA) before and after anastrazole treatment.

Patient	Response	PO-TC expr	rs6493497 polymorphism	rs7176005 polymorphism
*10054*	*NR*	*2.34*	*C/C*	*G/G*
**10043**	**R**	**2.33**	**C/T**	**G/A**
*10051*	*NR*	*1.18*	*C/C*	*G/G*
*10018*	*NR*	*0.17*	*C/C*	*G/G*
*10064*	*NR*	*0.16*	*C/C*	*G/G*
9999	R	0.13	C/C	G/G
10017	R	0.10	C/C	G/G
10010	R	0.06	C/C	G/G
*10000*	*NR*	*0.05*	*C/C*	*G/G*
***10028***	***NR***	***0.03***	***C/T***	***G/A***
10026	R	−0.51	C/C	G/G
10061	R	−0.92	C/C	G/G
***10056***	***NR***	−***1.03***	***C/T***	***G/A***
*10007*	*NR*	−*1.13*	*C/C*	*G/G*
*10052*	*NR*	−*1.67*	*C/C*	*G/G*
10013	R	−3.24	C/C	G/G

PO, post-operative; TC, before treatment; expr, aromatase RNA expression; R, responder; NR, non-responder. Polymorphic samples are in bold and non-responder tumors in italics.

**Table III.** CYP19A1 expression (log_2_ ratio of sample with respect to Human Universal Reference Total RNA) in TC sample RNAs.

Patient	Response	TC expr	rs6493497 polymorphism	rs7176005 polymorphism
10026	R	0.83	C/C	G/G
***10056***	***NR***	***0.72***	***C/T***	***G/A***
*10052*	*NR*	*0.25*	*C/C*	*G/G*
***10028***	***NR***	***0.22***	***C/T***	***G/A***
*10000*	*NR*	*0.17*	*C/C*	*G/G*
9999	R	0.15	C/C	G/G
10010	R	0.11	C/C	G/G
10017	R	0.09	C/C	G/G
10061	R	0.09	C/C	G/G
*10054*	*NR*	*0.02*	*C/C*	*G/G*
*10018*	*NR*	−*0.23*	*C/C*	*G/G*
*10051*	*NR*	−*0.25*	*C/C*	*G/G*
*10007*	*NR*	−*0.40*	*C/C*	*G/G*
*10064*	*NR*	−*0.58*	*C/C*	*G/G*
10013	R	−0.87	C/C	G/G
**10043**	**R**	−**3.56**	**C/T**	**G/A**

TC expr, before treatment aromatase RNA expression; R, responder; NR, non-responder. Polymorphic samples are in bold and non-responder tumors in italics.

**Table IV.** Statistical analysis of response to treatment and miRNA expression.

miRNA	Number of analyzed cases NR/R	Unpaired t-test P-value
miR-98	15/14	0.476503277
miR-1183	15/14	0.338253664
miR-1207-5p	15/14	0.714695237
miR-378	15/14	0.481222955
miR-661	15/14	0.187246281

NR, non-responder; R, responder.
